# Modeling, Control, and Optimization Technologies in Electric Drive Vehicles

**DOI:** 10.1155/2015/730347

**Published:** 2015-07-09

**Authors:** Hongwen He, Suleiman M. Sharkh, Caiping Zhang, Xuan Zhou, Rui Xiong

**Affiliations:** ^1^National Engineering Laboratory for Electric Vehicles, School of Mechanical Engineering, Beijing Institute of Technology, Beijing 100081, China; ^2^Collaborative Innovation Center of Electric Vehicles in Beijing, Beijing Institute of Technology, No. 5 South Zhongguancun Street, Haidian District, Beijing 100081, China; ^3^Electro-Mechanical Engineering Research Group, Engineering Science, University of Southampton, Highfield, Southampton SO17 1BJ, UK; ^4^School of Electrical Engineering, Beijing Jiaotong University, Beijing 100044, China; ^5^Electrical and Computer Engineering Department, Kettering University, Flint, MI 48504, USA

To address the two urgent issues nowadays of protecting the environment and achieving energy sustainability, it is of strategic importance on a global scale to replace oil-dependent vehicles with electric drive vehicles (EDVs). Numerical simulation and optimization are essential to simulate the actual hardware and minimize the development procedure and cost. Accurate and efficient modeling, control, and optimization technologies have been the indispensable tools. EDVs have been continuously improved owing to the advancement of systematic control and power management, energy systems, batteries, and pack technologies, as well as economic and policy incentives, public awareness of energy sustainability/affordability, and environmental concerns. The primary goal of this special section is to provide timely solutions to technological and economic challenges in modeling, simulation, control, and optimization of EDVs. The accepted papers cover a range of different aspects of modeling, control, and optimization technologies for electric vehicles. A brief summary of them is given as follows.

Most of papers are used to investigate the control and power management for EDVs. C. Lin and X. Cheng investigated a traction control strategy with an efficiency model in a distributed driving electric vehicle. S. Zhang et al. studied the optimal control strategy for a dual-motor coupling propulsion electric bus. The dynamic programming is applied to find the optimal control strategy including upshift threshold, downshift threshold, and power split ratio between the main motor and auxiliary motor. To improve the regenerative braking performance, J. Peng et al. provided a hierarchical control strategy for the cooperative braking system of an electric vehicle with separated driven axles. P. Lebeau et al. explored the possible integration of electric vehicles in urban logistics operations. A fleet size and mix vehicle routing problem with time windows for electric vehicles has been developed. In particular, an energy consumption model was integrated in order to consider variable range of electric vehicles. J.-J. Hwang et al. presented a novel charging strategy for fuel cell/battery electric vehicles. In comparison to the conventional switch control, a fuzzy control approach was employed to enhance the battery's state of charge (SOC).

In terms of the batteries and grouping technology, S. Barcellona et al. proposed a simple method to investigate the effect of the duty cycle on the batteries lifetime through tests performed on different cells for different kinds of cycle. In this way, a generic complex cycle could be seen as a composition of elemental cycles by means of Rainflow procedures. Consequently, the ageing due to any cycle could be estimated starting from the knowledge of simpler cycles from their research results. For the research field of energy systems and networks, L. Niu and D. Zhang analyzed the actual operational data of electric taxis in Shenzhen, China. An electric taxis charging guidance model was established and guides the charging based on the positions of taxis and charging stations with adaptive mutation particle swarm optimization. The simulation results showed that electric taxis could be evenly distributed to the appropriate charging stations according to the charging pile numbers in charging stations after the charging guidance.

In summary, the included seven papers cover the topics of modeling, control, and optimization technologies in EDVs with the new research achievements; we hope and are sure that the readers of this special issue could find valuable references to their research on EDVs.

